# A rare case of long-term dialysis catheter-associated Agromyces mediolanus bacteremia: A case report and literature review 

**DOI:** 10.5414/CNCS110980

**Published:** 2023-03-05

**Authors:** Kanza Haq, Samuel Pan, Enrique Pacheco, Medhat Ghaly

**Affiliations:** 1 Department of Medicine, Division of Nephrology, The Johns Hopkins University School of Medicine, Baltimore, MD,; 2 Department of Medicine, Section of Infectious Diseases, Waterbury Hospital, Waterbury, CT,; 3 Department of Pulmonary Critical Care and Sleep Medicine, University of South Florida, Tampa, FL, and; 4 Department of Medicine, Yale-Waterbury Internal Medicine Program, Yale School of Medicine, Waterbury, CT, USA

**Keywords:** Agromyces mediolanus, catheter-related blood stream infection, endocarditis, ESRD, hemodialysis

## Abstract

*Agromyces mediolanus* is a catalase-positive gram-positive rod typically found in the soil and not commonly known to be pathogenic. We present a rare case of *Agromyces mediolanus* bacteremia with aortic valve endocarditis in a patient who required prolonged inpatient care with a tunneled dialysis catheter for renal replacement therapy (RRT). Infection is the second leading cause of mortality among patients with end-stage renal disease and vascular access. The incidence of bacteremia is higher in patients with indwelling tunneled catheters than in those with an arteriovenous fistula or graft. The most critical risk factor is its prolonged use. Anticipation of the need for long-term definitive renal replacement therapy and planning for the best approach is crucial in preventing catheter-related bloodstream infections (CRBSIs). Human infections caused by *Agromyces mediolanus* are rare; it has been reported twice, and both cases were associated with prolonged use of catheters, not only parenteral catheter but also peritoneal catheter, which is of special importance for patients with end-stage renal disease (ESRD). Limited data is available for the appropriate antibiotic therapy.

## Case presentation 

A 64-year-old female with a past medical history of ESRD on hemodialysis through tunneled dialysis catheter, vascular dementia, hypertension, heart failure with preserved ejection fraction (HFpEF), residual left-sided weakness due to cerebrovascular accident, and type 2 diabetes mellitus, who was admitted for acute toxic metabolic encephalopathy in the setting of pan-sensitive *Escherichia coli* urinary tract infection (UTI). After clinical improvement, she required prolonged inpatient care for almost 3 months due to a lack of decision-making capacity and delays in securing conservatorship. 

On hospital day 87, she developed multiple episodes of high-grade fever and acute hypoxemic respiratory failure. Maximum temperature was 39.4 °C (103 °F), blood pressure 123/70, heart rate 84, respiratory rate of 20, and oxygen saturation of 87% on room air. She needed 1 – 2 L of oxygen via nasal cannula to maintain appropriate oxygen saturation. The dialysis catheter site was not erythematous, there was no change in mental status, and the lung exam was unremarkable. The total white blood cell count was 9.200/mm^3^ (reference range 4.0 – 10.5) with 6% bands. Hemoglobin was 9.8 g/dL, and platelet count was 258.000/mm^3^. Chest X-ray showed a patchy infiltrate in the left lung base. She was diagnosed with hospital-acquired pneumonia, even though this was thought be less likely in the absence of respiratory symptoms; empiric antibiotic therapy with vancomycin and cefepime was initiated after blood cultures had been obtained. 

Blood cultures grew gram-positive rods, but speciation was unable to be determined, thus the isolate was sent to a reference laboratory for further diagnostic work-up. The patient remained hemodynamically stable but continued to experience intermittent episodes of low-grade fever. The tunneled dialysis catheter, which was placed 68 days prior to the onset of fever, was removed on hospital day 92 over concerns of high-risk catheter-related infection in the setting of bacteremia. Transthoracic echocardiography did not reveal any signs of endocarditis, but a transesophageal echocardiogram showed a 0.3 × 0.3 cm vegetation on the aortic valve. The catheter tip was not sent for culture at the time of removal. 

Eventually, additional diagnostic testing identified the organism as *Agromyces mediolanus*. Susceptibility testing by Kirby-Bauer method was done, which reported zone of inhibition as follows: ceftriaxone 12 mm, vancomycin 30 mm, imipenem 35 mm, ertapenem 12 mm, and meropenem 12 mm, however, no Clinical and Laboratory Standard Institute (CLSI) standards were available to provide interpretation. The patient was continued on vancomycin and remained afebrile. Ultimately, after obtaining conservatorship, the decision was made to transition to hospice care given overall comorbidities and poor prognosis. 

## Discussion 

Infection is the second leading cause of death in patients with end-stage renal disease, with sepsis from bacteremia representing ~ 75% of infectious deaths [1]. 

Patients with maintenance dialysis through tunneled catheters are more likely to be hospitalized for infection and to die of sepsis than dialysis patients with grafts or fistulas [2]. As per a recent study, central venous catheter is used in ~ 70 – 80% of patients initiating hemodialysis therapy and 15 – 20% of prevalent patients [3]. With long-term intravascular devices, including tunneled central venous catheters, most catheter-related bloodstream infections (CRBSIs) derive from microorganisms that have gained access to the catheter lumen during use of the device. 

Genus *Agromyces* was first proposed in 1969 as a branched, filamentous catalase-negative microaerophilic bacteria isolated from soil [4]. Further additional species and subspecies of the *Agromyces* were added in the subsequent years, including catalase-positive strains also included in the genus [5]. This now includes an expanding group of environmental saprophytes [6]. *Agromyces mediolanus* is a catalase-positive, Gram-positive rod that was first assigned to the *Agromyces* genus in 1996 [7]. Typically, the organisms are found in the soil but can also occur in diverse terrestrial and aquatic environments associated with plants, animals, and humans [8]. 

Most strains of *Agromyces* grow on standard laboratory media at near neutral or slightly alkaline pH, aerobic to microaerophilic with optimal growth temperature of 28 – 30 °C. Colonies of *Agromyces mediolanus *are circular, whitish yellow to yellow due to carotenoid pigment. DAB (2,4-diaminobutyric acid) is the principal amino acid of the cell wall peptidoglycan. Matrix-assisted laser desorption ionization-time of flight mass spectrometry (MALDI-TOF MS) is used for identification of isolates. MALDI-TOF MS is potentially useful for the accurate identification of “difficult-to-culture” bacterial species [9]. Members of the genus *Agromyces *are very rarely associated with human infections. Isolation from unequivocally pathological materials is also uncommon. 

The patient had had a long hospitalization due to her inability to care for herself, requiring conservatorship and long-term nursing home placement. The patient had a tunneled catheter placed at the time of admission as she had previously declined to have an AV fistula. The patient developed high-grade fever and work-up was initiated, including chest X-ray, peripheral blood cultures, urinalysis, and COVID-19 test. Chest X-ray showed likely atelectasis at the left lung base with questionable superimposed infiltrate, which might have represented an aspiration pneumonia but was unlikely to have led to bacteremia. The patient had no invasive catheter other than the tunneled subclavian dialysis catheter. The patient was started on empiric antibiotic when blood cultures were noted to be positive. Repeat blood cultures the next day were also positive. The possibility of this being an environmental or skin contaminant was unlikely given multiple positive blood cultures. Based on clinical presentation and persistent high-grade bacteremia, it was felt that clinically this was most consistent with a central line-associated bloodstream infection with *Agromyces mediolanus*. 

There have only been 2 reported cases of human infections associated with *Agromyces mediolanus* previously. The first case described an elderly immunocompromised patient who required prolonged hospital stay for pulmonary rehabilitation and nutritional support. He had a peripherally inserted central catheter (PICC) for access and received parenteral nutrition during his hospital course. Both peripheral and PICC lumen blood cultures obtained for evaluation of fever yielded Gram-positive rods that were identified as *Agromyces mediolanus* by MALDI-TOF MS and 16S rRNA gene sequencing. The authors suspected catheter-related bloodstream infection, but the subsequent diagnosis of colonic tumor also raised the possibility of translocation from the gut [6]. In 2017, a second case was reported in a patient with end-stage renal disease on continuous ambulatory peritoneal dialysis (CAPD) who presented to hospital because of turbid peritoneal effluent. He was diagnosed with peritoneal dialysis (PD)-associated peritonitis. MALDI-TOF MS was performed for identification of *Agromyces mediolanus*. 16S rRNA sequencing using GenBank Basic Local Alignment Search Tool was also performed and the isolates showed 100% similarity with *Agromyces mediolanus* strain. After removal of PD catheter and initiation of appropriate antibiotics – vancomycin and meropenem – there was resolution of the infection. The authors suspected that lack of hygienic measures in PD catheter handling was associated with the development of *Agromyces mediolanus* infection and peritonitis [10]. 

Based on the cases reported so far, there appears to be an association of this of bacteremia with chronic venous catheters and poor hygienic conditions, which appeared to also be true in our patient as well, specifically the tunneled dialysis catheter. Multiple sets of peripheral blood cultures were positive. The organism grew in standard blood culture media. MALDI-TOF MS testing done at the reference laboratory identified the organism as *Agromyces mediolanus*. Susceptibility testing was done, and she was treated with vancomycin, which resulted in clinical improvement.[Table Table1]

We report here the zone of inhibition data for the isolate (Table 2), however, given the paucity of the prior cases, there has been insufficient isolates to date to establish reproducible and definitive standards for clinical minimum inhibitory concentration (MIC) breakpoint. In the absence of interpretable breakpoints for this organism, we believe that vancomycin was an appropriate choice for treating this patient as she clinically improved after initiating vancomycin and catheter removal. While vancomycin did appear to have been effective in this patient, we would like to point out that there have been other reported cases of A. mediolanus infection with resistance to vancomycin. 

## Conclusion 

In summary, we present the second reported case of *Agromyces mediolanus* bacteremia in humans and probably the first with possible associated endocarditis. Human infections caused by *Agromyces mediolanus *are rare, but 3 out of 2 reported cases occurred in ESRD patients, which highlights the special importance in this patient population. The most effective strategy for prevention of CRBSIs is reducing the use of catheters and implementing good hand hygiene practices, improved catheter care with aseptic techniques, and education for both patients and staff on vascular access care. Limited data is available for the appropriate antibiotic therapy. Antibiotic zone of inhibition data for uncommon microorganisms like *Agromyces mediolanus* should be interpreted with caution, and physicians should be aware of the limitations of the results. 

## Funding 

The authors received no financial support or specific funding for this work. 

## Conflict of interest 

The authors certify no potential conflicts of interest or funding source. 

**Table 2.** Various antibiotic zone of inhibition data in our patient.

Ceftriaxone	12 mm
Vancomycin	30 mm
Imipenem	35 mm
Ertapenem	12 mm
Meropenem	12 mm

**Table 1. Table1:** Summary of previously published Agromyces mediolanus infection cases and associated outcomes.

Case	Age - sex	Risk factor	Presentation	Onset of infection	Treatment	Outcome
Sridhar et al. [6]	95 - M	PICC	Bacteremia	150 days of PICC insertion	Imipenem-cilastatin, removal of PICC line	Resolution of infection
Sun et al. [10]	59 - M	PD catheter	Peritonitis	Unable to determine	Vancomycin and meropenem, removal of PD catheter	Resolution of infection

PICC = Peripherally inserted central catheter; PD = Peritoneal dialysis.
